# Globular Adiponectin Activates Motility and Regenerative Traits of Muscle Satellite Cells

**DOI:** 10.1371/journal.pone.0034782

**Published:** 2012-05-18

**Authors:** Tania Fiaschi, Elisa Giannoni, Maria Letizia Taddei, Paola Chiarugi

**Affiliations:** Department of Biochemical Science, University of Florence, Florence, Italy; Childrens Hospital Los Angeles, United States of America

## Abstract

Regeneration of adult injured skeletal muscle is due to activation of satellite cells, a population of stem cells resident beneath the basal lamina. Thus, information on soluble factors affecting satellite cell activation, as well as migration towards injury and fusion into new myofibers are essential. Here, we show that globular adiponectin (gAd), positively affects several features of muscle satellite cells. gAd activates satellite cells to exit quiescence and increases their recruitment towards myotubes. gAd elicits in satellite cells a specific motility program, involving activation of the small GTPase Rac1, as well as expression of Snail and Twist transcription factors driving a proteolytic motility, useful to reach the site of injury. We show that satellite cells produce autocrine full length adiponectin (fAd), which is converted to gAd by activated macrophages. In turns, gAd concurs to attract to the site of injury both satellite cells and macrophages and induces myogenesis in muscle satellite cells. Thus, these findings add a further role for gAd in skeletal muscle, including the hormone among factors participating in muscle regeneration.

## Introduction

Adiponectin is a intensely studied hormone due to its ability to control glucose and lipid homeostasis and to have anti-atherogenic and anti-inflammatory properties [1]. The hormone is secreted as “full-length” (fAd) form that is cleaved by neutrophil elastase, generating the smaller “globular” (gAd) form. Although it has been largely reported that gAd has considerable biological effects on different tissues such as liver, skeletal muscle and endothelium, the generation of gAd is still debated. Both fAd and gAd binds, although with different affinity, to the two atypical seven membrane spanning receptors AdipoR1 and AdipoR2 [2].

gAd increases glucose uptake in cultured myocytes or isolated muscle cells, and alters lipid metabolism through the stimulation of muscle fatty acid oxidation [2]; [3]. Recently, we suggested a new role of gAd in skeletal muscle, showing its involvement in the regeneration of dystrophic muscles. We reported that gAd induces myogenesis in cultured myoblasts and enhances muscle differentiation of mesoangioblasts, a multipotent non-resident precursor muscle cells. The treatment of mesoangioblasts with gAd protects them from *in vivo* apoptosis, increasing their engraftment in the *tibialis anterior* of dystrophic mice [4]; [5].

Muscle regeneration is a very complex process involving both resident and non-resident cells with myogenic properties. Among non-resident precursors, a variety of different cells having myogenic properties have been isolated, including adipose tissue-derived stem cells [6], mesoangioblasts [7], pericytes [8] muscle derived stem cells [9], side-population cells [10]–[12], Ac133^+^ cells [13], stem and/or precursor cells from muscle endothelium [14] and sinovium [15]. In healthy muscle upon injury, these cells are attracted in the site of damage where they can differentiate or fused with pre-existing myofibers. The major participants in adult muscle regeneration are muscle satellite cells (mSAT) which reside underneath the basal lamina of mature muscle fibers. After the trauma, mSAT shift from quiescence to the activated state, proliferate and differentiate to generate new fibers. It has been reported that the activation/phosphorylation of p38 MAPK is a key step for mSAT activation/exit from the quiescence. Indeed, inhibition of p38MAPK induces their exit from cell cycle and prevent differentiation [16]. Activation of p38 MAPK by adiponectin has been already reported for hematopoietic stem cell proliferation, proposing this adipokines as a stem cell factor for these cells [17].

Based on these previous observations, we investigated the role of adiponectin in mSAT. We show that gAd, produced by both mSAT and macrophages, has a pleiotropic effect in mSAT inducing their migration to the site of injury, finally promoting muscle differentiation. These results suggest a new role of adiponectin in skeletal muscle as stem cell factor and propose a new function of the hormone in this tissue in addition to its well-known metabolic activities.

## Materials and Methods

### Materials

Unless specified all reagents were obtained from Sigma except PVDF membrane (Millipore), anti-muscle Myosin Heavy Chain (mMHC), anti-AdipoR1, anti-AdipoR2, anti-Snail, anti-Twist, anti-vimentin, anti-adiponectin and anti-actin antibodies AdipoR1 shRNA Lentiviral Particles and AdipoR2 shRNA Lentiviral Particles, (Santa Cruz), anti-phospho-p38 (Thr180/Tyr182), anti-p38 (Cell Signalling), anti-Rac1 antibodies (BD Transduction Laboratories). gAd and fAd were from Alexis, Alexa 488 fluorescent secondary antibodies was from Molecular Probes. Diff-Quik staining kit was from Medion Diagnostics. Amicon Ultra-Centrifugal filter Units were from Millipore. RNeasy mini kit, Quantitech reverse transcription Kit and Quantifast SYBER Green PCR were from Qiagen.

**Figure 1:**
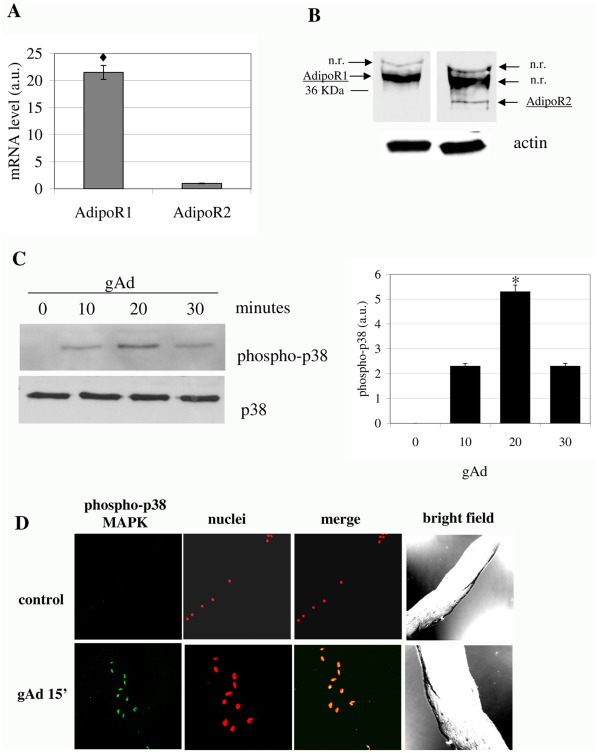
gAd induces the activation of mSAT. **A**) Analysis by Real-time PCR of AdipoR1 and AdipoR2 expression on mSAT. The amount of target was normalized to the endogenous reference (β2-microglobulin) and was obtained by the 2^ΔΔCT^ calculation. Data are reported as arbitrary units (a.u.), ♦p<0,001; **B**) Western blot analysis using AdipoR1 and AdipoR2 specific antibodies. Actin immunoblot is shown below to assure equal protein loading; n.r: non-related band. **C**) p38 MAPK in mSAT. Isolated mSAT were serum deprived for 18 hours and then stimulated with gAd (1 µg/ml) for the indicated period. Detection of p38 MAPK phosphorylation was obtained by anti-phospho-p38-MAPK immunoblot followed by p38-MAPK immunoblot for normalization. The bar graph represents the quantification of three independent experiments. *p<0,01; **D**) Freshly isolated fibers were stimulated with gAd (1 µg/ml) for 15 minutes. The fibers were fixed and treated with anti-phospho-p38-MAPK antibodies and with propidium iodide to label nuclei. p38-MAPK phosphorylation was detected using 488 Alexa Fluor-conjugated secondary antibodies. All blots are representative of four independent analyses.

### Cell Cultures

mSAT were obtained by 5–10 weeks old wtC57BL/6J mice killed by cervical dislocation. *Tibialis anterior* muscles were isolated and digested in 0,2% collagenase as previously described [18]. The experiment was carried out in accordance with national guidelines and approved by the ethical committee of Animal Welfare Office of Italian Work Ministry and conform to the legal mandates and Italian guidelines for the care and maintenance of laboratory animals. Myofibers and associated satellite cells were seeded in Matrigel (1 mg/ml) and cultured in plating medium (DMEM supplemented with 10% horse serum (HOS), 0.5% chicken embryonic extract, 4 mM L-glutamine and 1% penicillin-streptomycin) at 37°C in 5% CO_2_. mSAT were removed by enzymatic treatment with 0.25% trypsin-EDTA solution for 10 minutes at 37°C and maintained in high-serum-containing medium (DMEM supplemented with 20% FBS, 1% chicken embryo extract, 10% HOS, 4 mM L-glutamine and 1% penicillin-streptomycin). For myogenic differentiation, mSAT were cultured in differentiation medium (DMEM containing 2% HOS) for four days at 37°C. Human Embryonic Kidney (HEK) cells and C2C12 murine myoblasts were cultured in DMEM supplemented with 10% Fetal Bovine Serum, 4 mM L-glutammine and 1% penicillin-streptomycin, at 37°C in 5% CO_2_. Myotubes used in migration assay were obtained by culturing C2C12 murine myoblasts in the lower chamber of the Boyden assay and treated with differentiation medium for four days. Murine macrophages RAW 294.7 were cultured in DMEM supplemented with 10% FBS at 37°C in 5% CO_2_.

**Figure 2:**
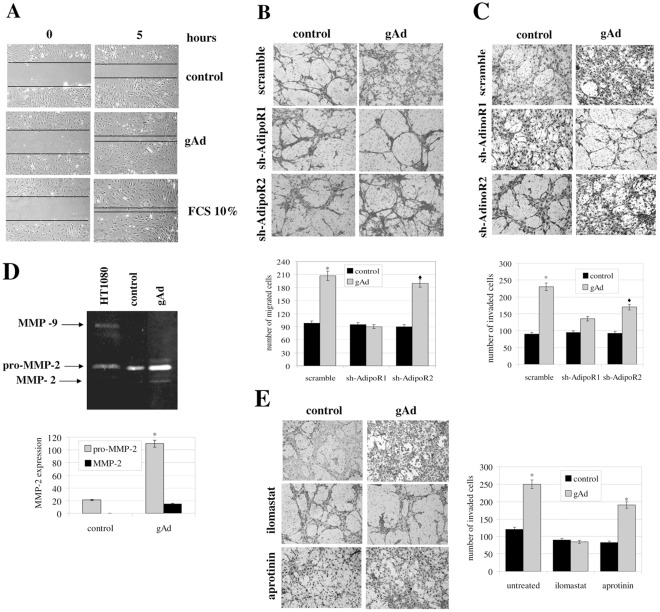
gAd enhances the motility of mSAT. **A**) Wound healing assay. Confluent mSAT were serum deprived overnight and then we performed an artificial wound. Cells were treated with free-serum medium (control), free-serum medium containing gAd (1 ug/ml) or 10% FCS. Cells were photographed after 5 hours. Black lines are indicative of the remaining gap in the wound. **B**) Migration of mSAT induced by gAd. mSAT were serum deprived overnight and then seeded in the upper chamber of the Boyden assay in free-serum medium with or without gAd (1 µg/ml). Four days differentiated C2C12 myotubes were cultivated in the lower chamber and used as chemo-attractant, *p<0,001 *vs* control. **C**) The invasiveness assay was performed as described in B, except for the presence of a thin layer of Matrigel in the upper chamber, *p<0,001 and ♦p<0,01 *vs* control. **D**) gAd enhances the production of MMP-2. mSAT were treated for 18 h with serum-free medium with or without gAd (1 µg/ml) and the corresponding media were subjected to zymography analysis. *p<0,001 *vs* control. **E**) The invasiveness assay of mSAT in response to gAd was performed as in C, except for the presence of the Ilomastat (broad MMP inhibitor) or aprotinin (uPA inhibitor), *p<0,001 and ♦p<0,01 *vs* control. Representative images of migrated mSAT, after staining with hematoxylin-eosin, are shown in B, C and E. Bar graphs represent the mean of migrated cells counted in six different fields for each experiment.

### Real-Time PCR

Total RNA was isolated using RNeasy mini kit. One microgram of total RNA was used for reverse transcription using Quantitech reverse transcription Kit according to manufacturer’s protocol. Measurement of gene expression was performed by quantitative Real-Time PCR (7500 Fast Real Time PCR System, Applied Biosystems) using Quantifast SYBER Green PCR. The amount of target was normalized to β2-microglobulin as endogenous reference. The primer sets for mouse AdipoR1/R2 were as follows:

mouse AdipoR1 forward primer: 5′ACGTTGGAGAGTCATCCCGTAT;

mouse AdipoR1 reverse primer: 5′CTCTGTGTGGATGCGGAAGAT;

mouse AdipoR2 forward primer: 5′TCCCAGGAAGATGAAGGGTTTAT;

mouse AdipoR2 reverse primer: 5′TTCCATTCGTTCGATAGCATGA

### Western Blot

Cells were lysed for 10 min on ice in complete c-RIPA lysis buffer (0,1% SDS, 0,5% deoxycholate, 50 mM Tris-HCl, pH 7.5, 150 mM NaCl, 1% Nonidet P-40, 2 mM EGTA, 1 mM sodium orthovanadate, 1 mM phenyl-methanesulphonyl-fluoride, 10 µg/ml aprotinin, 10 µg/ml leupeptin). Lysates were clarified by centrifugation. An equal amount of each sample was run on SDS/PAGE and transferred onto PVDF membrane. Immunoblots were performed as already described [19] and analysed by a Kodak Gel Logic 2200 for dedicated chemiluminescent image acquisition.

**Figure 3:**
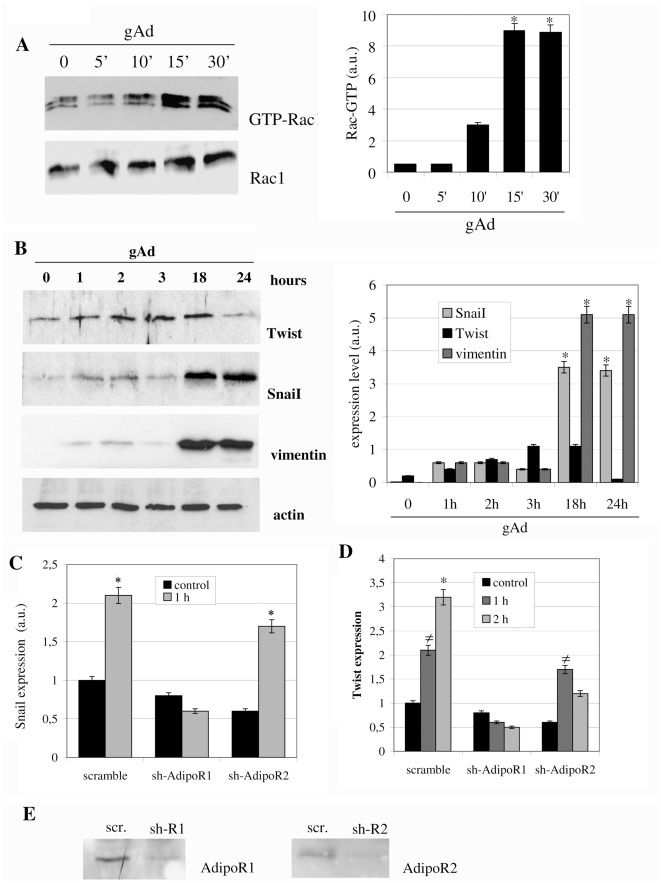
gAd activates a mesenchymal motility program in mSAT. mSAT were treated overnight with free-serum medium and then stimulated with gAd (1 µg/ml) for the period indicated. **A**) Rac-1 activation assay. 50 µg of total proteins were used in the GST pull down assay for Rac-1 and the amount of GTP-bound Rac-1 was detected by anti-Rac-1 immunoblot after the pull down. Normalization has been carried out by anti-Rac-1 immunoblot analysis of cell lysates. Normalized data are shown in the bar graph. *p<0,001 *vs* time 0. **B**) Expression of mesenchimal markers Twist, Snail and vimentin. 20 ug of total proteins were loaded in each lane and the expression level of Snail-1, Twist and vimentin was analysed by immunoblots. Anti-actin immunoblot was used for normalization and the normalized data are reported in the bar graph, *p<0,001 *vs* time 0. **C**)**, D**) mSAT were permanently transduced with AdipoR1 (C) or AdipoR2 (D) shRNAs and treated as described in B), *p<0,01 and #p<0,025 *vs* time 0. **E**) Expression analysis of AdipoR1 and AdipoR2 in mSAT after transduction with AdipoR1 or AdipoR2 shRNAs. All immunoblots are representative of three different and independent experiments.

### Immunofluorecence

Satellite cells were cultured on glass cover slips in growing medium. For immunofluorescence cells were washed with PBS and fixed in 3% paraformaldehyde for 20 min at 4°C. Fixed cells were permeabilized with three washes with T-PBS (PBS containing 0,1% Triton X-100) and then blocked with 5,5% horse serum in T-PBS for 1 h at room temperature. Cells were then incubated with specific primary antibodies, diluted 1∶100 in PBS, overnight at 4°C. Cells were then washed once with T-PBS and once with T-PBS with 0,1% BSA and incubated with secondary antibodies (diluted 1∶100) for 1 hour at room temperature in T-PBS with 3% BSA. After extensive washes in T-PBS cells were mounted with glycerol plastine and observed with a confocal fluorescence microscope (Leica TCS SP5). For immunofluorescence of muscle fibers, freshly isolated fibers were washed in PBS, fixed in 4% paraformaldehyde and then treated as described above.

**Figure 4:**
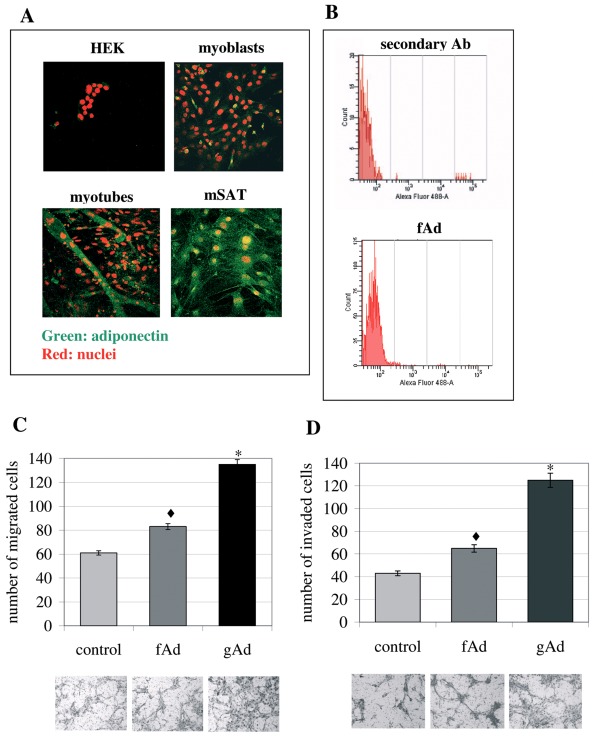
mSAT are a source of autocrine fAd. A) Analysis of adiponectin expression in Human Embryonic Kidney (HEK) cells, C2C12 murine myoblasts, four days differentiated C2C12 and mSAT by confocal microscopy. Cells were seeded on cover slip, fixed and then treated with anti-adiponectin antibodies and with propidium iodide to label the nuclei. The visualization of adiponectin was performed using Alexia Fluor 488-coniugated secondary antibodies. The images are representative of four independent experiments. **B**) Analysis of adiponectin expression by citofluorimetric analysis. Cells were treated as in A) and then analyzed using a FACSCanto cytofluorimeter. **C**) Migration of mSAT induced by fAd and gAd. mSAT were serum deprived overnight and then seeded in the upper chamber of the Boyden assay in free-serum medium supplemented with fAd (1 µg/ml) or gAd (1 µg/ml). Four days differentiated C2C12 myotubes were cultured in the lower chamber and used as chemo-attractant. **D**) The invasiveness assay was performed as described in C, except for the presence of a thin layer of Matrigel in the upper chamber. Images are representative of four independent experiments and the mean of migrated cells in six independent experiments was shown in the bar graphs. *p< 0,002 and ♦p<0,01 *vs* control.

### Wound-healing Assay

Confluent satellite cells were serum deprived overnight and then a scratch on the monolayer was performed with sterile tips. Cells were then treated with serum-free medium with or without gAd (1 µg/ml) or with medium containing 10% FCS used as positive control. The cells were photographed immediately after making the artificial wound (time 0) and at the end of the experiment.

**Figure 5:**
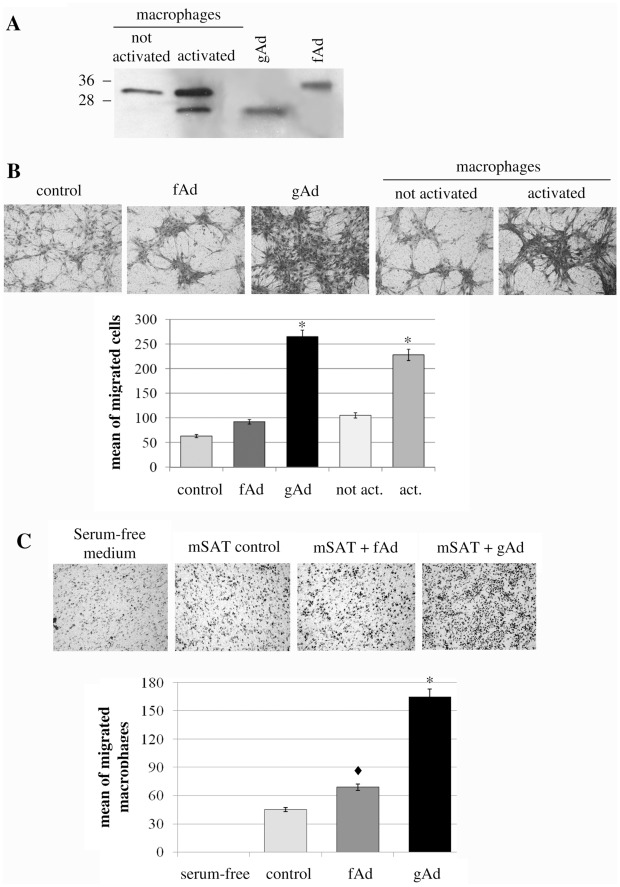
Activation of macrophages leads to fAd proteolysis and gAd production. **A**) Activated murine macrophages secrete gAd. RAW 294.7 Macrophages are activated by incubation for 24 h with free-serum medium contained LPS (10 ng/ml) and IFN-γ (50 U/ml). Culture media were concentrated 20-fold and analysed by anti-adiponectin immunoblot. Recombinant fAd and gAd were used in parallel as markers. **B**) Conditioned media from activated macrophages induces the migration of mSAT towards differentiated myotubes. After activation, murine RAW 294.7 macrophages were treated with serum-free medium for 24 hours to remove LPS and INF-γ and then the corresponding conditioned media were used for mSAT migration assay towards myotubes. mSAT were serum deprived overnight and then seeded in the upper chamber of the Boyden assay in free-serum medium with or without exogenous fAd (1 µg/ml) or gAd (1 µg/ml) or in resting or activated macrophage-conditioned media. Four days differentiated C2C12 myotubes were cultured in the lower chamber and used as chemoattractant. *p<0,001 *vs* control. **C**) Adiponectin attracts macrophages towards satellite cells. Murine RAW 294.7 macrophages were serum deprived overnight and then seeded in the upper chamber of a Boyden assay in serum free medium. mSAT were cultured in the lower chamber in free serum medium with or without fAd (1 µg/ml) or gAd (1 µg/ml) and used as chemoattractant. Migrated macrophages were detected after 16 hours by hematoxylin-eosin staining. Images are representative of four independent experiments with similar results. *p<0,001 and ♦p<0,01 *vs* control.

### Migration Assay

For migration assay, 3×10^4^ satellite cells (serum deprived overnight) were seeded in the upper chamber of transwell (membrane diameter: 6,5 mm; pore size: 8 µm) in serum-free medium with or without fAd (1 µg/ml) or gAd (1 µg/ml). For invasion assay, the filter of the transwell was coated with Matrigel and 1×10^5^ satellite cells were plated in the upper chamber. Four days differentiated C2C12 cells were cultured in the lower chamber of transwells and placed in serum-free medium during the migration assay. The number of satellite cells migrated through the membrane pores was evaluated after 16 hours. Non-migrated cells were mechanically removed from the upper side of the transwell system, whereas the cells in the lower side of the filter membrane were colored using Diff-Quik Staining Kit according the manufacturer’s instruction. The number of migrated cells was obtained by counting 3–5 random fields of the lower face of the transwell membrane at 10 X magnification.

**Figure 6:**
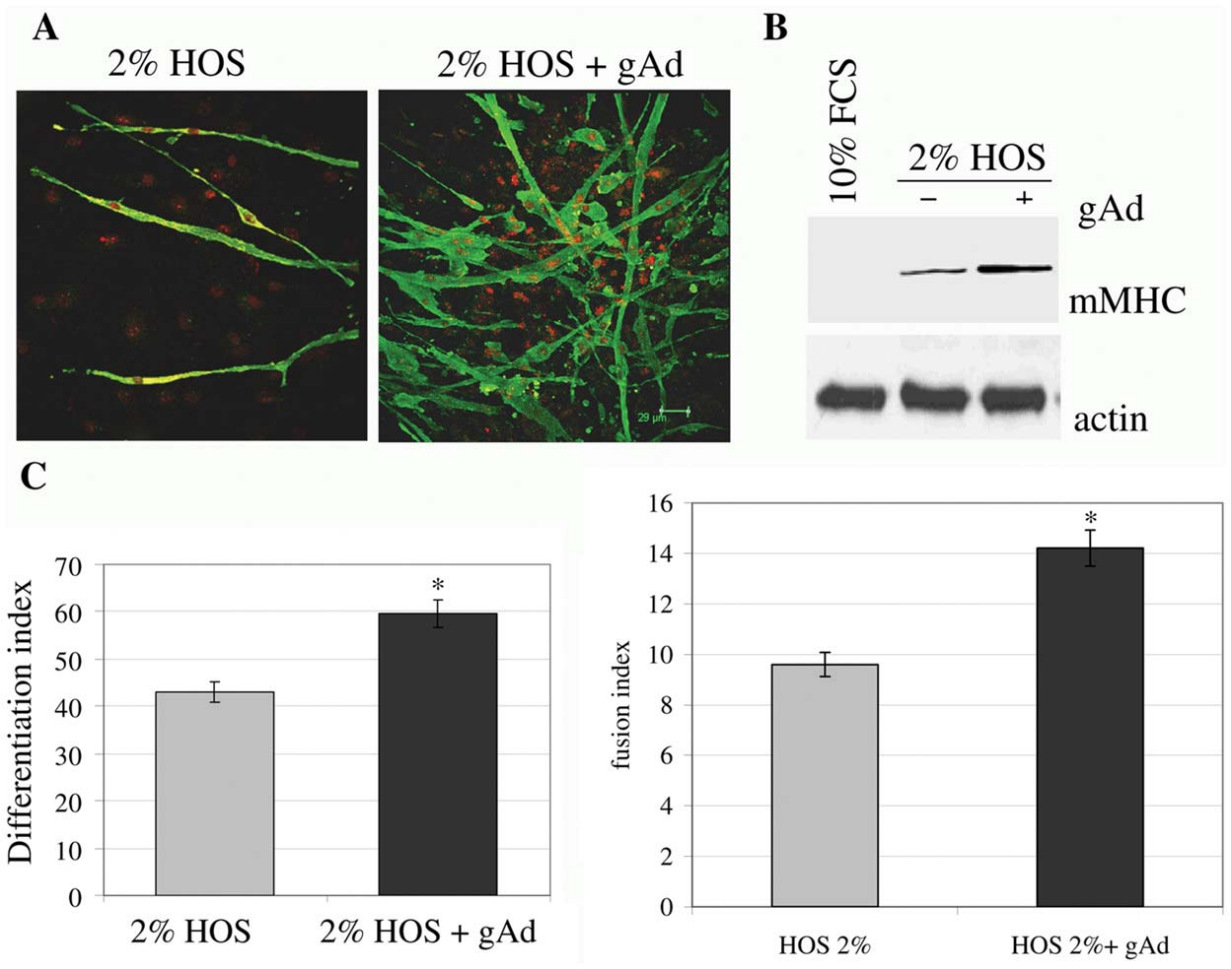
gAd enhances myogenesis of mSAT. Subconfluent mSAT were cultured for four days in differentiating medium (DMEM containing 2% HOS) with or without gAd (1 µg/ml). **A**) Cells were labeled with anti-mMHC primary antibodies and then with Alexa Fluor 488-coniugated secondary antibodies (green). Nuclei were stained with propidium iodide (red). These images are representative of three independent experiments with similar results. **B**) Analysis of mMHC expression was performed in four days differentiated cells using anti-mMHC antibodies. Actin immunoblot was used for normalization. **C**) Differentiation index and **D**) fusion index of four days differentiated cells, calculated as reported in the Materials and Methods section. *p<0,01 *vs* 2% HOS.

### Metalloproteinase Zymography

Aliquots from media in our experimental conditions were run on 8% SDS-PAGE co-polymerized with 0.1% (w/v) type A gelatin. Gels were washed twice in 2.5% v/v Triton X-100 for 30 minutes and then incubated in 50 mM Tris-HCl, pH 7.4, 200 mM NaCl and 5 mM CaCl_2_ at 37°C for 24 hours. After incubation, the gels were stained with 0.1% Coomassie brilliant blue in acetic acid, methanol, and distilled water (1∶2∶3, respectively) for 60 minutes at room temperature. After destaining, the gels were immersed in distilled water and scanned immediately with Quantity-One Image Analysis software (Bio-Rad). Bands of gelatinase activity appeared as transparent areas against a blue background.

**Figure 7:**
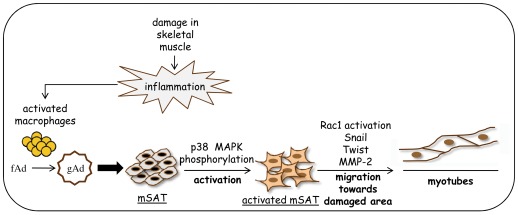
Proposed model of gAd action in skeletal muscle. After mucle damage, recruited/activated macrophages induce the proteolytic conversion of fAd to gAd, which in turn elicites p38 MAPK phosphorylation, thus participating in mSAT activation. Furthermore, gAd promotes mSAT migration towards the damaged site through the induction of a mesenchymal motily program. This mechanism provides the activation of the small GTPase Rac1, the *de-novo* expression of Snail and Twist transcription factors and the increased expression of MMP-2, mandatory to allow mesenchymal motility. After reaching the site of damage, gAd induces mSAT differentiation culminating in the formation of new myotubes and contributing to regeneration.

### RNAi Against AdipoR1 and AdipoR2

50% confluent satellite cells were infected with 15 µl of AdipoR1 or AdipoR2 shRNA lentiviral particles according to manufacturer’s instruction. 48 hours after infections cells were serum deprived and used for experiments.

### Muscle Differentiation

Freshly isolated mSAT were differentiated in differentiating medium (DMEM containing 2% HOS) with or without gAd (1 µg/ml) for four days. Differentiation index was calculated as the ratio between mMHC-positive cells and total nuclei and the fusion index as the average number of nuclei in muscle MHC-positive cells containing at least three nuclei above the total nuclei.

### Activation of RAW 294.7 Macrophages

5×10^6^ macrophages were treated with serum-free medium containing LPS (10 ng/ml) and IFN-γ (50 U/ml) for 24 h. The medium was then concentrated by centrifugation at 4000 rpm for 1 h and then used for further analysis. Amicon Ultra-Centrifugal filter Units were used to concentrate the media.

### Statistical Analysis

Data are presented as means±S.D. from at least three experiments. Analysis of densitometry was performed using Quantity One Software (Bio-Rad). Statistical analysis of the data was performed by Student’s *t*-test. *P*-values ≤0,05 were considered statistically significant.

## Results

### 1. gAd Induces p38 MAPK Phosphorylation in mSAT

After isolation of mSAT form the TA of wt C57BL/6 mice, we firstly analyzed the expression of adiponectin receptors, AdipoR1 and AdipoR2. Results of both Real Time PCR and immunoblot analyses reveal in both cases that AdipoR1 is more expressed with respect to AdipoR2 (Fig. 1A and 1B).

It has been reported that p38 MAPK reversibly controls the exit from cell cycle and the maintenance of quiescent state of satellite cells, thus supporting the idea that p38MAPK acts as a molecular switch for activation of satellite cells [16]. Based on our previous results on myoblasts and mesoangioblasts [4]; [5], we wondered whether gAd induces the p38 MAPK phosphorylation in satellite cells. We observed that gAd promotes p38 MAPK phosphorylation with a maximum peak at 20 minutes after stimulation (Fig. 1C). Similar results were obtained by confocal microscopy analysis of intact fibers from TA, after their treatment for 15 minutes with gAd (Fig. 1D), thereby suggesting that gAd could be involved in muscle satellite cell activation.

### 2. gAd Increases the Motility of Satellite Cells Towards Myotubes

Muscle regeneration requires both the activation of satellite cells and their migration at the site of damage. Hence, knowledge of the factors that increase the motility of these cells is essential. Firstly, we found that gAd increases cell migration towards myotubes of about two folds with respect to control and repairs the artificial wound similarly to the positive control, containing 10% FCS (Fig. 2A, 2B and Fig. S1). The same pro-migratory effect by gAd was observed when cells are forced to overcome a barrier of extracellular matrix (ECM), as in matrigel-coated Boyden assay (Fig 2C). Activation of the motility program specifically involves AdipoR1, as the silencing of this receptor greatly reduces the motility of the cells towards myotubes, while the silencing of AdipoR2 has a marginal effect (Fig. 2B and 2C). In keeping with its pro-invasive effect, gAd enhances the production of metalloproteinase (MMP)-2 by mSAT (Fig. 2D) which is essential for mSAT migration, as the inhibition of MMP-2 activity with ilomastat almost completely abolishes the migration of cells. In contrast, the use of aprotinin, an inhibitor of the urokinase-type plasminogen activator (uPA) proteolytic system, does not produce effects on the motility of these cells (Fig. 2E).

### 3. gAd Activates a Motility Program in mSAT

Cell migration is a general process involving different mechanisms in different cell types and tissue environments. These specific programs are driven by defined signalling molecules that could shift from inactive to active state (as Rac/Rho small GTPases), and by the *de-novo* expression of other proteins, as specific transcription factors and surface molecules as cadherins or MMPs [20]. Firstly, we checked the activation of the small GTPase Rac1, acknowledged to be involved in mesenchymal migration, the most common motility style for polarized cells [21]. We observed a significant activation of Rac1 following gAd administration, which starts 15 minutes after stimulation and remains unchanged thereafter, in keeping with an activation of polarized motility of satellite cells (Fig. 3A).

Mesenchymal motility is commonly driven by *de-novo* synthesized transcription factors, such as Snail-1 and Snail-2, Twist or the ZEB family [20]. We therefore performed a time-course experiments to check the expression of the transcription factors Snail-1 and Twist in mSAT following gAd stimulation. We observed that gAd induces an earlier increase of Twist expression, peaking at two hours of treatment and remaining unchanged up to 18 hours, followed by expression of Snail-1 until 24 hours (Fig. 3B). Of note, activation of these transcription factors leads to a specific enhancement of the motile phenotype, as indicated by the strong increase in the expression of vimentin. (Fig. 3B). Again gAd mainly acts through its AdipoR1 receptors, as indicated by RNA interfering with AdipoR1/R2 receptors. Indeed, the expression of both Snail-1 and Twist upon gAd administration is decreased by silencing of AdipoR1, while the silencing of AdipoR2 has only a marginal effect (Fig. 3C–D).

### 4. mSAT are a Source of Autocrine fAd

Although adiponectin has been firstly described to be produced mainly by adipose tissue, other sources have been reported, including endothelium, pituitary gland, liver and differentiated myotubes [22]; [23]. We therefore analyzed whether mSAT produce adiponectin. Both confocal microscopy and cytofluorimetric analyses show that these cells produce the full-length form of the hormone (Fig. 4A-B).

On the basis of production of fAd by mSAT, we compared fAd and gAd efficiency in inducing the migration of mSAT. Our results show that fAd causes only a slight increase of the motility of mSAT towards myotubes, confirmed for both migration and invasiveness (Fig. 4C and 4D). The lower effect of fAd with respect to gAd in inducing a motile phenotype in mSAT suggests that a specific proteolysis of fAd is likely needed to maximize adiponectin efficacy at the site of injury.

### 5. Activation of Macrophages Leads to fAd Proteolysis and gAd Production

Nevertheless it is not clear how and where gAd is produced, it is extensively reported that the cleaved hormone has several specific biological effects on different tissues such as liver, skeletal muscle and endothelium [3]; [22]; [24]. The group of Kadowaki reported that the monocytic cell line THP-1 express the enzyme elastase that *in vitro* cleaves fAd generating three different forms of gAd of apparent molecular weight of 25, 20 and 18 KDa [25]. We tested whether murine RAW 294.7 macrophages produce factors able to cleave fAd. Murine macrophages were either activated with lipopolysaccarides (LPS) and interferon-γ (IFN-γ) or maintained inactivated for 24 h. Their conditioned media were then analyzed by anti-adiponectin immunoblot. We observed that RAW 294.7 macrophages actively secrete fAd. In addition, after their activation, macrophages secrete a shorter form of the hormone, corresponding to the 25 KDa gAd form (Fig. 5A), thus pointing on activated macrophages as a possible source of gAd during inflammation.

Conditioned media from activated and resting macrophages were used in a migration assay in which mSAT were induced to migrate towards myotubes. As expected, conditioned media from resting macrophages (containing autocrine fAd) induces only a scarse migration of mSAT, similar to the effect induced by exogenously administered fAd (Fig. 5B). On the contrary, conditioned medium from activated macrophages (containing autocrine gAd) elicits a very strong motility, comparable with the effect induced by exogenous gAd (Fig. 5B).

Several studies demonstrated that macrophages, that are recruited to the site of injury, play an important role in muscle regeneration. In particular, macrophages promote the growth of mSAT, protecting them from the apoptotic stimuli and increasing their differentiation [26]. The presence of macrophages appears crucial for muscle regeneration since their depletion prevents correct muscle recovery as well as the development of fibrosis and muscle impairment [27]. Thus, knowledge of the factors that attract macrophages at the site of damage is of particular importance. We then examined whether fAd and gAd promote the migration of macrophages towards mSAT. Figure 5C shows that macrophages actively migrate towards mSAT and that the treatment with gAd and fAd enhances this effect, being gAd two-fold more active with respect to the full length hormone.

### 6. gAd is a Myogenic Factor for mSAT

Based on our previous observations that gAd acts as myogenic factor both in cultured myoblasts and in mesoangioblasts [4]; [5], we investigated the ability of gAd to induce muscle differentiation in mSAT. We observed that the administration of gAd to standard differentiation medium (HOS 2%) increased the expression of murine myosin heavy chain (mMHC), as shown both by confocal microscopy and immunoblot analyses (Fig. 6A–B). In addition, gAd increases both the differentiation and the fusion indexes, thus demonstrating that gAd acts as a pro-myogenic factor even in mSAT (Fig. 6C–D).

## Discussion

Data presented herein demonstrated that gAd affects several features of mSAT, the master resident muscle stem cells, with the final aim to induce myogenesis. We found that: i) gAd activates a mesenchymal motility/invasive program; ii) mSAT and macrophages produce autocrine fAd; iii) gAd is produced by activated macrophages through proteolytic cleavage of fAd; iv) gAd induces the migration of macrophages towards mSAT and then v) gAd promotes muscle differentiation of mSAT, thereby completing this positive loop.

In healthy muscles, satellite cells are quiescent and show a low expression of a small subset of growth factor-related genes including fibroblast growth factor (FGF) receptors 1 and 4 and c-Met/hepatocyte GF (HGF) [28]. After damage, satellite cells became immediately activated, begin to proliferate and induce the expression of myogenic regulatory factors [29]. The passage from quiescent to activated state is a fundamental step for satellite cells. It has been reported that activation of satellite cells requires the activation/phosphorylation of p38 MAPK and that its inhibition prevents MyoD induction, thus blocking satellite cells activation and proliferation [16]. In keeping, we reported that gAd induces a strong activation of p38 MAPK, both in intact fibers and in isolated satellite cells, thus pointing on gAd as a candidate factor participating in mSAT cell activation following a trauma.

Another key step during regeneration of adult skeletal muscle is the migration of satellite cells towards the injured site [30]. Following the trauma, activated satellite cells migrate to the site of damage and differentiate to form new fibers that fuse to each other or fuse to local surviving fibers, contributing to muscle regeneration. During this process cells must degrade the extracellular matrix surrounding muscle fibers, through the production of specific MMPs. MMPs have been reported to be important players in migration and differentiation of muscle cells [31]–[34]. Several studies indicated the important role of MMP-2, −7 and −9 in myotubes formation, due to their capacity to eliminate ECM and thus permitting the fusion between two cell membranes. In particular, MMP-2 plays a key role in muscle regeneration, being up-regulated during the first three days following trauma and enhancing their activity concomitantly with the regeneration of the new myofibers [30]; [35]. In agreement, we found that gAd increases the migration of satellite cells towards myotubes and enhances the production of MMP-2, thus permitting the degradation of ECM. The increased migration of satellite cells induced by gAd is accompanied by the activation of specific program correlated with mesenchymal motility [20], such as activation of the small GTPase Rac1, the increased expression of the transcription factors Snail-1 and Twist, as well as the mesenchymal marker vimentin. The expression/activation of these proteins occurs in most tissues and drives the acquisition of migratory properties. For example, the expression of these proteins during embryogenesis or in cancer cells takes place during the so-called “epithelial-mesenchymal transition” (EMT). Although muscle cells do not meet EMT, we assess that gAd increases mesenchymal properties of mSAT enhancing their migration and invasiveness. We already reported that in hepatic cells gAd induces the activation of Rac1 leading to reactive oxygen species production, which are indispensable for the down-stream metabolic effects induced by the hormone [19]. In mSAT Rac1 activation has already been involved in rearrangement of the cytoskeleton, involving actin polymerization and lamellipodia formation [21]. For what the transcription factors Twist and Snail is concerned, studies of their involvement in muscle regeneration are still very preliminary. To date, only Snail-1 has been reported as a regulator for the emergence of muscle progenitors from dermomyotomo [36], while the function of Twist in skeletal muscle is totally unknown. Here, we involve both transcription factors in the gAd-driven acquisition of motile phenotype of satellite cells towards differentiated myotubes.

In addition to adipose tissue, where the hormone has been firstly detected, adiponectin is produced in many other districts such as cardiomyocytes, differentiated myotubes, osteoblasts, placenta, endothelium and pituitary cells [22]. In particular, we showed that myotubes generate adiponectin in an autocrine manner and that this production is further enhanced by inflammatory cytokines [4]. Here, we show that mSAT produce fAd, a less effective hormone with respect to gAd in inducing their motility. It has been reported that fAd and gAd have different biological activities and that *in vitro* gAd is generated by proteolytic cleavage of fAd by neutrophil elastase [3]; [24]; [37]–[39]. Although gAd is abundant in human plasma, the exact location of gAd generation and the meaning of this production remains to be defined. We now reported that inflammation may be a key event in gAd proteolysis.

Beyond the mere removal of debris, several observations suggest a key role for macrophages in promoting muscle repair and remodeling after injury. For example, transplantation of myogenic cells is impaired if monocytes and macrophages were depleted by irradiation in the recipients [40]. In addition, conditioned media from cultures of peritoneal macrophages or monocyte cell lines increase the proliferation of myoblasts *in vitro* and enhance the number of MyoD-expressing cells [41]. Furthermore, it has been reported that activated satellite cells use macrophages to escape apoptosis and enhance muscle growth [26]. Hence, the identification of macrophage-derived factors that promote muscle repair *in vivo* is an important question to be answered. We found that murine RAW 294.7 monocytes basally produce fAd, which is cleaved into gAd following macrophage activation. In keeping with an active proteolysis of fAd from activated macrophages, their conditioned medium shows a chemo-attractive effect of mSAT comparable to exogenous gAd and not to fAd. These findings support the idea that activated macrophages constitute a source of gAd, more efficient than fAd in inducing activation and migration of mSAT towards the damaged muscle region.

To induce their beneficial effects, macrophages need to be attracted in the site of injury. It has been reported that mSAT produce monocyte chemoattractants such as monocyte/macrophage-derived chemokine, monocyte chemoattractant protein-1, fractalkine and vascular endothelial GF [26]. Of note, we now show that gAd enhances the ability of mSAT to attract macrophages, being gAd more efficient than fAd, thereby suggesting that gAd plays a pleiotropic role during muscle regeneration, through the recruitment of macrophages as well as the activation and migration of mSAT (Fig. 7).

Beyond its metabolic role, our previous results clearly suggested an additional function of adiponectin in skeletal muscles as myogenic factor. We show that gAd is a strong inducer of myogenesis in C2C12 myoblasts and *in vivo* promotes the survival and the engraftment of mesoangioblasts in skeletal muscle of dystrophic mice [4]; [5]. Again, we observed that gAd is a powerful myogenic factor in mSAT due to its ability to enhance myogenesis.

Overall, our findings propose gAd as a powerful and pleiotropic hormone for skeletal muscle, involved in the regulation of metabolic pathways, as well as in the regeneration of damaged/diseased tissue. gAd behaves as a stem cell factor acting on different muscle precursors, including resident mSAT and non- resident endothelial precursors recruited to damaged/diseased muscles as mesoangioblasts. On this basis, gAd can therefore serve as a new tool for treating muscle diseases for which stem cell therapies have been proposed, as congenital or acquired myopathies or severe post-injury atrophy.

## Supporting Information

Figure S1 **Wound healing assay by time lapse live microscopy**. mSAT were serum-deprived overnight and an artificial wound was made. Cells were then treated with free-serum medium (control) or free-serum medium containing gAd (1 ug/ml). Time-lapse recordings were performed on a Zeiss Televal 31 inverted microscope with 10× achromatic objective coupled to a Panasonic wv-BP330 CCD camera. Phase contrast images of cells were collected consecutively every 30 sec for various durations ranging from 18 hours. Images were edited using Animator DV software. Representative images of control or gAd-treated cells were shown.
(DOC)
