# Supplementary figures and images for: Globular Adiponectin Activates Motility and Regenerative Traits of Muscle Satellite Cells
Source: PLoS One. 2012 May 18;7(5):e34782. doi: 10.1371/journal.pone.0034782 (PMC3356356; doi:10.1371/journal.pone.0034782)

**Figure S1.**


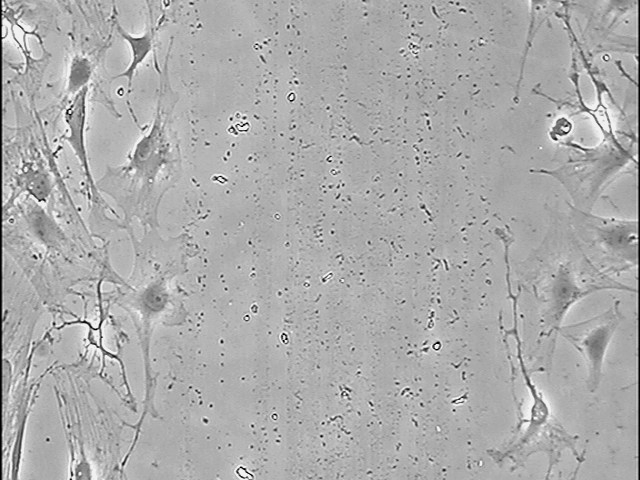

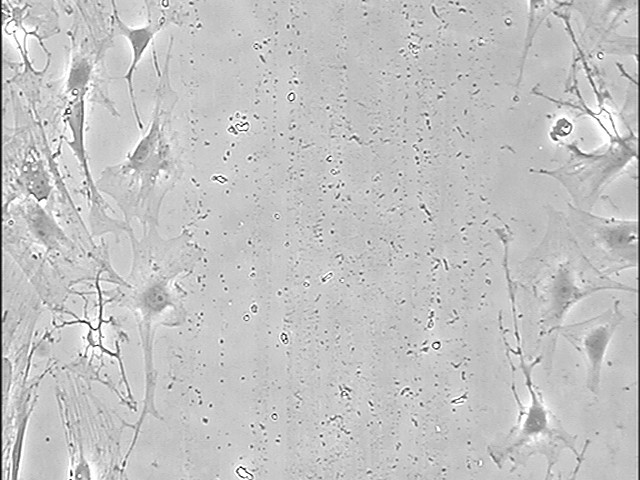

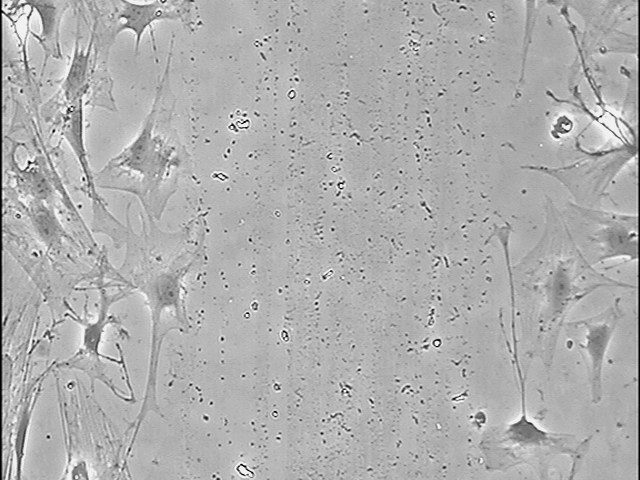

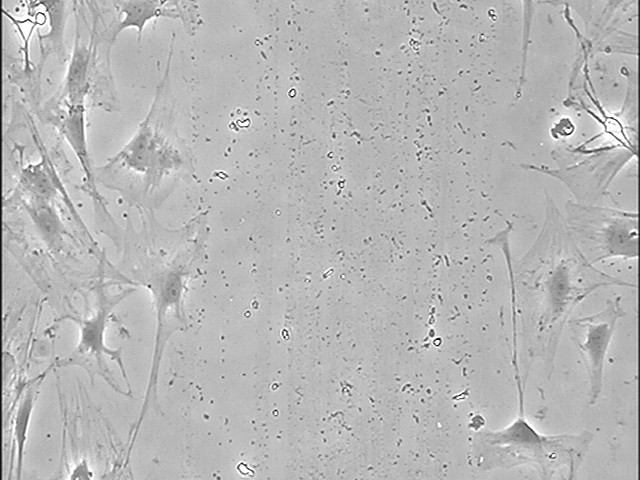

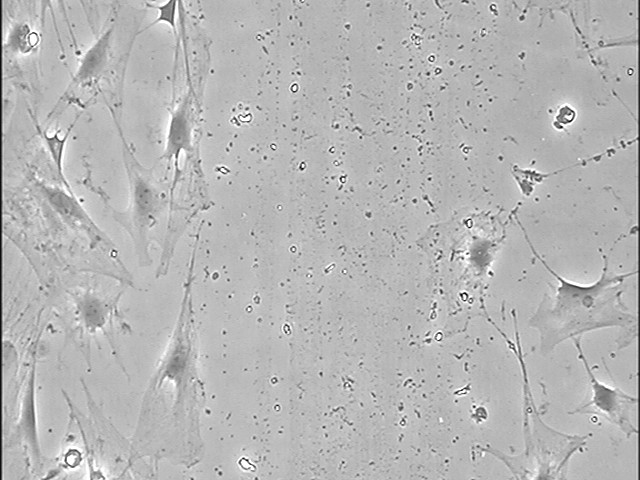

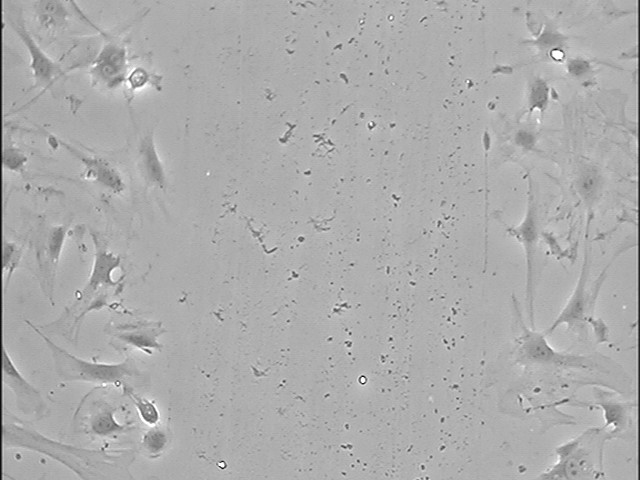

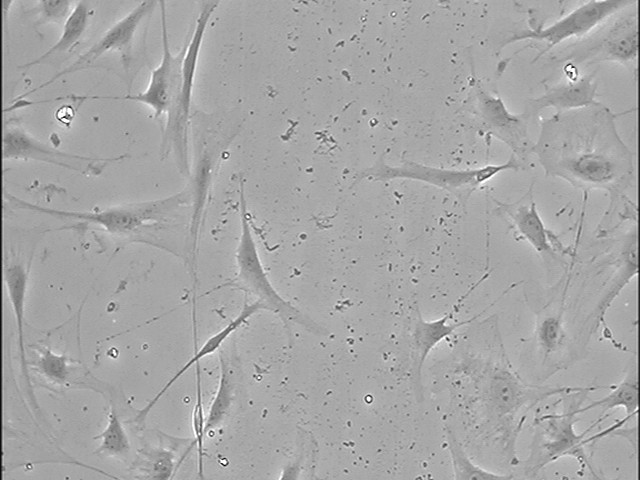

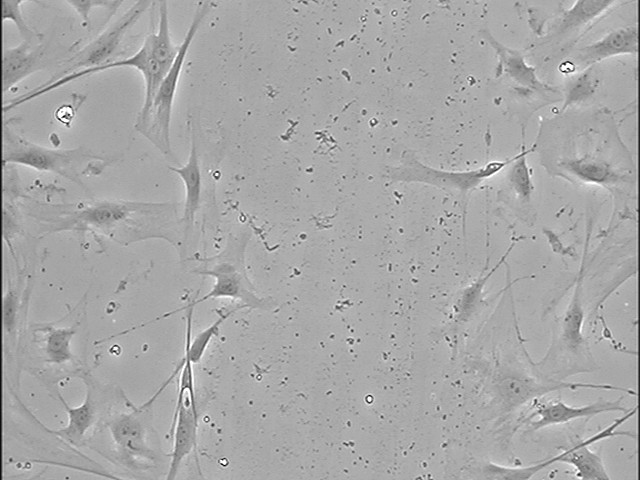

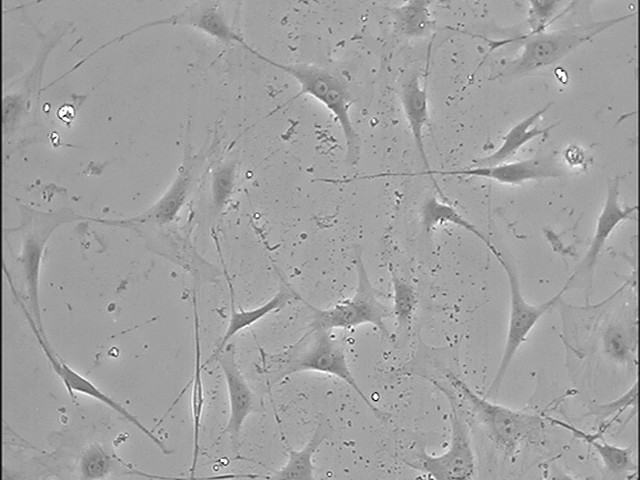

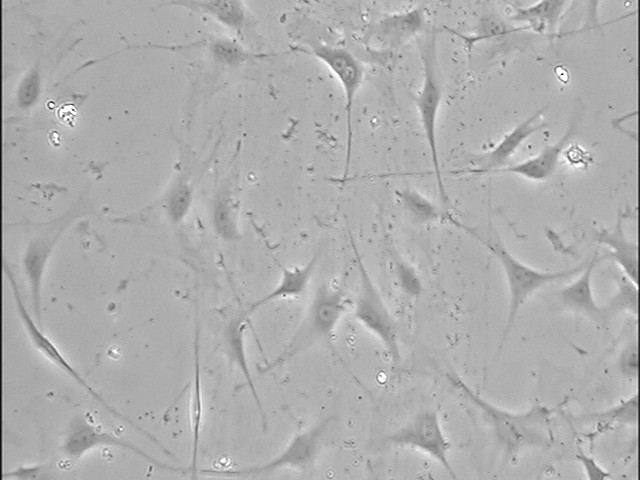

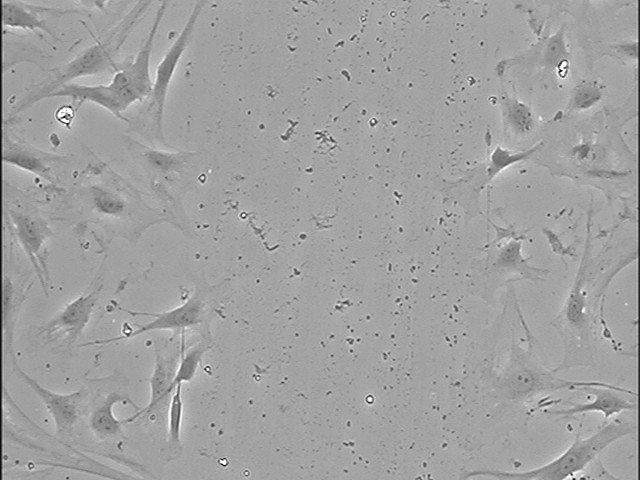


18 h

15 h

12 h

9 h

6 h

3 h

control

gAd

Supplement: Figure S1 — Wound healing assay by time lapse live microscopy. mSAT were serum-deprived overnight and an artificial wound was made. Cells were then treated with free-serum medium (control) or free-serum medium containing gAd (1 ug/ml). Time-lapse recordings were performed on a Zeiss Televal 31 inverted microscope with 10× achromatic objective coupled to a Panasonic wv-BP330 CCD camera. Phase contrast images of cells were collected consecutively every 30 sec for various durations ranging from 18 hours. Images were edited using Animator DV software. Representative images of control or gAd-treated cells were shown. (DOC) [file pone.0034782.s001.doc]
